# The impact of a superfood chocolate beverage on indices of peripheral vascular function

**DOI:** 10.14814/phy2.70448

**Published:** 2025-07-03

**Authors:** John O. Kolade, R. Matthew Brothers

**Affiliations:** ^1^ Department of Kinesiology The University of Texas at Arlington Arlington Texas USA

**Keywords:** blood pressure, cardiovascular disease, lipid profile, polyphenols, vascular function

## Abstract

Elevated blood lipids and reduced vascular health contribute to cardiovascular disease. Diets and/or interventions high in antioxidants, polyphenols, and anti‐inflammatory properties mitigate this risk. This study hypothesized that daily consumption of a beverage high in antioxidants, polyphenols, and anti‐inflammatory properties would improve vascular function, blood lipids, and arterial blood pressure. Vascular function was assessed as brachial artery flow mediated vasodilation (%FMD) and following 5 min of ischemia. Brachial artery blood pressure was assessed, and blood samples were collected for lipid analysis. Measures were assessed in 15 individuals free from cardiovascular or other diseases (5 male; age: 33 ± 12 years) before (Pre) and 2 weeks following (Post) consumption of the Goode Health Superfood Moroccan Chocolate Blend (~28 g of powder added to 8 oz water twice daily). This beverage is rich in plant‐based proteins, prebiotic fiber, antioxidant polyphenols, and anti‐inflammatory properties. %FMD was augmented following 2 weeks of beverage consumption (Pre: 4.7 ± 2.1 vs. Post: 5.8 ± 2.2%, *p =* 0.04). Mean blood pressure (Pre: 89 ± 5 vs. Post: 86 ± 6 mmHg, *p =* 0.01) and total cholesterol (Pre: 174 ± 26 vs. Post: 159 ± 25 mg/dL, *p =* 0.04) were lower following 2 weeks of beverage consumption. These data indicate a positive effect of the Goode Health beverage on indices of vascular endothelial function, blood lipids, and arterial blood pressure.

## 1 INTRODUCTION

Cardiovascular disease (CVD) remains the most prominent source of morbidity and mortality in the United States (Martin et al., 2024). Numerous factors contribute to the development and presence of CVD; however, reductions in vascular endothelial function/health, characterized by elevated vascular resistance and arterial wall stiffness, and reduced vasodilator function, are primary contributors (Vanhoutte et al., 2017; Widmer & Lerman, 2014). Impaired endothelial function/health results in a myriad of conditions including exercise intolerance, impaired thermoregulatory function, and is a precursor to or coexists with various cardiovascular, metabolic, and neurocognitive pathophysiological conditions including hypertension, atherosclerosis, coronary heart disease, type II diabetes, stroke, cognitive decline, and Alzheimer's disease and other related dementias (ADRD) (Dede et al., 2007; Hughes et al., 2015, 2018; Vanhoutte et al., 2017; Widmer & Lerman, 2014).

Lifestyle interventions, particularly those targeting diet and exercise, are effective in preventing, maintaining, or improving many of these pathophysiological conditions (Ghodeshwar et al., 2023; Rippe, 2019; Rippe & Angelopoulos, 2014). Often, a common link among these interventions is their ability to enhance vascular endothelial function (Cerqueira et al., 2019; Gao et al., 2022; Ghodeshwar et al., 2023; Nystoriak & Bhatnagar, 2018; Trombetta et al., 2003). Extensive evidence from cross‐sectional studies, acute and long‐term interventions, and epidemiological research indicates that diets, beverages, and/or supplements containing high antioxidant, polyphenol, and anti‐inflammatory properties are inversely correlated with hypertension, atherosclerosis, coronary artery disease, stroke incidence, and ADRD (Arts & Hollman, 2005; Balzer et al., 2008; Barbour et al., 2014; Djousse et al., 2006; Heiss et al., 2005, 2010; Hurr et al., 2015; Iqbal et al., 2023; Sanches‐Silva et al., 2020; Santhakumar et al., 2018; Schroeter et al., 2006). Furthermore, these improvements are particularly notable in populations with reduced vascular endothelial function (Balzer et al., 2008; Grassi et al., 2008; Heiss et al., 2005; Hurr et al., 2015; Park et al., 2010). Conversely, there is evidence to suggest that too high of an antioxidant dose can actually limit beneficial adaptations to perturbations including exercise (Li et al., 2022). The proposed mechanisms of these beneficial effects are multifactorial but are related to improvements in antioxidant capacity and thus reduced oxidative stress, anti‐inflammatory properties, and subsequent increased nitric oxide (NO) bioavailability as well as reduced lipid peroxidation and thus improvements in blood lipid profile (Arts & Hollman, 2005; Balzer et al., 2008; Barbour et al., 2014; Djousse et al., 2006; Heiss et al., 2005, 2010; Hurr et al., 2015; Iqbal et al., 2023; Liu, 2003; McCullough et al., 2012; Park et al., 2010; Sanches‐Silva et al., 2020; Santhakumar et al., 2018; Schroeter et al., 2006). Importantly, these physiological processes are associated with improved indices of vascular endothelial function.

Goode Health, https://www.goodehealth.com/, is a commercial provider of products made with polyphenols and other plant‐based ingredients. However, whether increased daily consumption of these products improves well‐validated indices of peripheral vascular endothelial function remains unclear. Accordingly, we performed a randomized, double‐blind, placebo‐controlled study to test the hypothesis that daily consumption of Goode Health Moroccan Chocolate Blend (twice daily for 2 weeks) would improve macrovascular and microvascular endothelial function as indexed by brachial artery flow‐mediated dilation (FMD) and forearm reactive hyperemia (RH) respectively, and blood lipid profiles, in a cohort of relatively young and otherwise healthy individuals.

## 2 METHODS

### 2.1 Ethical approval

All procedures were approved by the Institutional Review Board at the University of Texas at Arlington (UTA IRB: 2023‐0202). Participants were given a verbal description of all procedures, purposes, and risks before providing their informed, written consent. This study conformed to the Declaration of Helsinki and was a registered clinical trial under the identifier NCT06245382 at www.clinicaltrials.gov.

### 2.2 Participant characteristics

Fifteen individuals (10 women) participated in the experimental beverage portion of the study and 16 individuals (six women) participated in the control beverage portion (Table 1). See below for detailed information about the beverages. All participants were normotensive, free of overt cardiometabolic or other diseases, nonsmokers, not currently taking vitamin or mineral‐based supplements, and were recreationally active. Participants were excluded if they were taking vasoactive medications, had overt cardiovascular, metabolic, or neurological disease, or were participating in competitive athletic activities.

**TABLE 1:** Participant characteristics.

Variable	Control	Experimental	*p* Value
	Beverage group (*n* = 16)	Beverage group (*n* = 15)	
Men/Women	10/6	5/10	–
Age (years)	23 ± 6	33 ± 12	*0.01*
Height (cm)	171 ± 11	167 ± 11	0.45
Weight (kg)	76.2 ± 14.9	84.4 ± 25.6	0.36
BMI (kg/m^2^)	26.2 ± 4.7	30.0 ± 7.2	0.16

*Note*: Data are represented as means ± SD.

Abbreviation: BMI, body mass index.

### 2.3 Instrumentation and measurements

All data were collected following a minimum 4 h fast and prior to each data collection visit participants refrained from strenuous exercise and caffeinated or alcoholic beverages for a minimum of 24 h and all data were collected in a temperature‐controlled laboratory (∼24°C and 40% relative humidity). Phase of the menstrual cycle was recorded but not controlled. Upon arrival at the laboratory, participants voided their bladder, and then height and body mass were acquired using a digital scale and standard stadiometer (Seca 769, Seca North America; Chino, CA).

After lying semi‐recumbent on a laboratory bed, each participant was instrumented for continuous measures of heart rate (electrocardiography; CardioCard, Nasiff Associates; Central Square, NY) and intermittent blood pressure of the brachial artery via an electrosphygmomanometer (Tango+, SunTech; Raleigh, NC).

#### 2.3.1 Venous blood draw

Blood was drawn via venipuncture into serum separator tubes. Samples were subsequently centrifuged at room temperature for 10 min at 3400 revolutions per minute (Laboratory Corporation of America, Model 642e Centrifuge, Drucker Diagnostic). After centrifugation, serum was analyzed for blood lipid profile within 24 h at a local laboratory (Laboratory Corporation of America, Burlington, NC).

#### 2.3.2 Brachial artery flow‐mediated dilation and forearm reactive hyperemia

Following 15 min of supine rest in which heart rate was continuously assessed and brachial artery pressure was measured in triplicate, measures of macrovascular and microvascular function, brachial artery FMD, and forearm RH, respectively, were performed as previously described by our group and others (Akins et al., 2022; Martin, Akins, et al., 2023; Martin et al., 2022; Martin, Al‐daas, et al., 2023; Patik, Tucker, et al., 2018; Thijssen et al., 2019). Briefly, the right arm was abducted and supported at heart level while a pneumatic cuff was wrapped around the forearm, ~1 cm distal to the antecubital fossa. The brachial artery was insonated ~5–10 cm proximal to the antecubital fossa using an adjustable frequency (7–12 MHz) linear array Doppler ultrasound probe (LOGIQ P5; GE Healthcare; Chicago, IL, USA), held in place by a stereotactic probe holder at an angle of 60°. B‐mode images of the brachial artery were optimized to ensure delineation between the vessel wall and lumen during offline analysis. The probe was then set to duplex mode which allowed for imaging of the artery and blood velocity. Throughout this data collection period the sample volume was adjusted to include the entire lumen of the artery. Continuous measures of brachial artery diameter (*D*; cm) and blood velocity were recorded during 2‐min baseline. After the baseline period the cuff was rapidly inflated to 220 mmHg to induce forearm ischemia for 5 min (Rapid Cuff Inflation System; D. E. Hokanson, Inc.; Bellevue, WA, USA). After this 5‐min period the cuff was released and measures continued for an additional 3‐min. Throughout this protocol the images were recorded and saved (Elgato/CORSAIR; Fremont, CA, USA) for offline analysis using edge‐detection and velocity tracking software (FMD Studio; Quipu; Pisa, Italy).

### 2.4 Experimental beverage condition

The aforementioned measurements were assessed at baseline and after 2 weeks of daily consumption of the Goode Health Moroccan Chocolate Blend (Table 2). Following the baseline data collection visit the participants were provided 28 separate Ziploc bags, each containing ~28 g of Moroccan Chocolate Blend powder. Participants were instructed to mix the contents of each bag with 8 oz. of water and consume the beverage mix twice daily (once in the morning and again in the evening) for a total of 14 days. Each bag was clearly labeled (e.g., “Day 1 AM,” “Day 1 PM,” “Day 2 AM,” “Day 2 PM,” etc.) to support adherence. Following the 14‐day intervention, participants returned to the laboratory for their post‐intervention data collection visit. Importantly, to control for the potential circadian impact on the measured physiological variables, pre‐ and post‐beverage consumption data collection for each condition was conducted at the same time of day (±30 min) within each individual.

**TABLE 2:** Experimental and control beverage ingredients.

	Control beverage (% daily value)	Experimental beverage (% daily value)
Calories	80	80
Total fat	1.5 g (2%)	2.5 g (3%)
Saturated fat	0 g (0%)	0 g (0%)
Trans fat	0 g (0%)	0 g (0%)
Cholesterol	0 mg (0%)	0 mg (0%)
Sodium	45 mg (2%)	40 mg (2%)
Total carbohydrate	9 g (3%)	7 g (3%)
Dietary fiber	1 g (4%)	5 g (18%)
Total sugars	1 g (0%)	0 g (0%)
Protein	15 g (28%)	14 g (28%)

*Note*: Other ingredients. Control beverage: Rice protein blend, maltodextrin, natural flavors, stevia leaf extract, and monk fruit extract. Experimental beverage: Pea protein, rice protein, fava bean, chia seed, berberine, monk fruit, cinnamon, bergamot, bilberry, echinacea, green tea, ginger, black garlic, meriva turmeric, cocoa, flax seed, vitamin blend (magnesium, potassium, calcium, vitamin A, vitamin B9, vitamin C, vitamin D, and vitamin E).

### 2.5 Control beverage condition

The aforementioned measurements were also assessed at baseline and following 2 weeks of daily consumption of a vanilla protein blend control beverage (Table 2). Importantly, this product was formulated to replicate commonly available vegetable protein powders that are available in the market but lacked the polyphenols and anti‐inflammatory and antioxidant properties present in the experimental beverage.

### 2.6 Participant compliance

For both the experimental and control beverage conditions, the participants were instructed to maintain their typical dietary and physical activity behaviors. Compliance during both conditions was encouraged, monitored, and maximized via the following procedures: (1) frequent email/text message communication to remind the individuals to consume the beverages; (2) the participants returned their empty bags on the post‐intervention data collection visit; and (3) the participants completed a daily beverage consumption log which was also returned during their post‐intervention data collection visit.

### 2.7 Data analysis

During each study visit, resting brachial blood pressure was measured in triplicate (⅓ systolic + ⅔ diastolic) following 15 min of quiet supine rest. Brachial artery %FMD was defined as $\left(\left(D\text{peak}-D\text{baseline}\right)/D\text{baseline}\right)\times 100$. RH was calculated as the peak forearm mean blood velocity (FBVmean) measured after cuff release.

### 2.8 Statistical analysis

Participant characteristics were compared using unpaired, two‐tailed *t*‐tests. Baseline hemodynamics, blood metabolic parameters, and peripheral vascular function were analyzed using two‐way mixed‐effects design analysis of variance (ANOVA) with the main factors of beverage condition (i.e., experimental and control) × time (i.e., pre and post consumption). *T*‐tests were performed in the event of a significant interaction. All data were processed using GraphPad Prism 8 (v. 8.4.3; GraphPad Software, LLC, San Diego, CA, USA). Alpha was set at *p =* 0.05.

## 3 RESULTS

### 3.1 Participant characteristics and baseline

Participant characteristics are presented in Table 1. The group that consumed the Goode Health Moroccan Chocolate Blend beverage was slightly older (*p =* 0.01); otherwise, the groups were well matched for height, weight, and BMI (*p* > 0.16 for all). Likewise, the baseline pre‐intervention values for heart rate, systolic blood pressure (SBP), diastolic blood pressure (DBP), mean blood pressure (MAP), as well as lipid profile were also similar between groups (Table 3). Both beverage conditions demonstrated similar compliance rates, with the participants consuming ~83% of the provided doses.

**TABLE 3:** Pre‐ and post‐beverage consumption hemodynamic and blood lipid variables for each condition.

Variable	Control beverage group		Experimental beverage group		*p* Value
	Pre	Post	Pre	Post	
HR (bpm)	63 ± 9	65 ± 10	68 ± 12	64 ± 12	Beverage: 0.71 Time: 0.74 Beverage × Time: 0.20
SBP (mmHg)	122 ± 7	119 ± 7	119 ± 6	116 ± 7	Beverage: 0.31 Time: <0.001 Beverage × Time: 0.92
DBP (mmHg)	72 ± 7	70 ± 8	74 ± 5	72 ± 5	Beverage: 0.41 Time: 0.01 Beverage × Time: 0.76
MAP (mmHg)	89 ± 6	86 ± 7	89 ± 5	87 ± 6	Beverage: 0.85 Time: <0.01 Beverage × Time: 0.87
TC (mg/dL)	164 ± 22	167 ± 17	174 ± 26	159 ± 29^a^	Beverage: 0.92 Time: 0.13 Beverage × Time: 0.03
TG (mg/dL)	64 ± 18	64 ± 23	75 ± 23	65 ± 20	Beverage: 0.50 Time: 0.21 Beverage × Time: 0.14
HDL (mg/dL)	54 ± 11	57 ± 13	49 ± 9	48 ± 9	Beverage: 0.13 Time: 0.36 Beverage × Time: 0.11
VLDL (mg/dL)	13 ± 3	13 ± 4	14 ± 3	14 ± 4	Beverage: 0.32 Time: 0.89 Beverage × Time: 0.89
LDL (mg/dL)	98 ± 20	97 ± 15	111 ± 21	98 ± 22	Beverage: 0.39 Time: 0.08 Beverage × Time: 0.13

*Note*: Data are represented as means±SD.

Abbreviations: DBP, diastolic blood pressure; HDL, high‐density lipoprotein; HR, heart rate; LDL, low‐density lipoprotein; MAP, mean arterial blood pressure; SBP, systolic blood pressure; TC, total cholesterol; TG, triglycerides; VLDL, very low‐density lipoprotein.

^a^ Total cholesterol was significantly reduced in the experimental condition but was unaffected by the control beverage.

### 3.2 Vascular endothelial function

**FIGURE 1:**
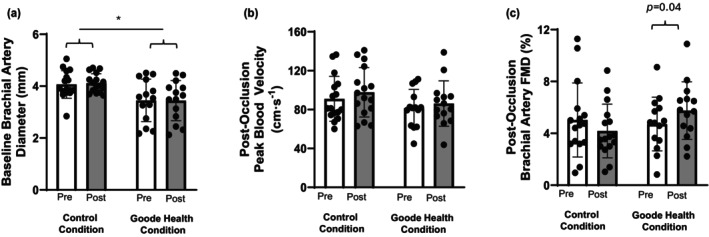
Baseline brachial artery diameter (Panel a), microvascular function indexed as peak blood velocity (reactive hyperemia) following cuff occlusion induced ischemia (Panel b), and macrovascular function as indexed by brachial artery flow‐mediated dilation (Panel c) before (white bars) and following (gray bars) 2‐weeks of consumption of the control beverage (Control Condition) and the Goode Health Moroccan Chocolate Blend (Goode Health Condition). Diameter and peak blood velocity were not impacted by either beverage condition whereas flow‐mediated dilation was significantly elevated in the experimental condition but was unaffected by the control beverage. *Significant group effect where baseline diameters were larger in the control condition (*p =* 0.008). Data are presented individually and as group means ± SD.

Baseline pre‐intervention values for brachial artery diameter were slightly larger in the control group (Figure 1a; *p =* 0.02), whereas baseline pre‐intervention values for microvascular function indexed as RH (peak forearm mean blood velocity following cuff release; Figure 1b, *p =* 0.23) and macrovascular function indexed as FMD (Figure 1c, *p =* 0.73) were similar between groups. While brachial artery diameter remained slightly larger in the control group following both beverage consumption conditions (Figure 1a; main effect of beverage condition, *p* = 0.008) there was no effect of time (Figure 1a; pre vs. post, *p* = 0.73), nor was there a condition × time interaction (Figure 1a; *p* = 0.65). There was no effect of beverage condition (Figure 1b; *p* = 0.18) or time (Figure 1b; pre vs. post; *p* = 0.10), nor was there a condition × time interaction (Figure 1b; *p* = 0.78) on RH. There was a significant condition × time interaction for brachial artery FMD (Figure 1c; *p* = 0.04) such that FMD was improved following 2 weeks of consumption of the Goode Health Moroccan Chocolate Blend (Figure 1c; Pre: 4.7 ± 2.1%, Post: 5.8 ± 2.2%, *p =* 0.04).

### 3.3 Blood lipids and arterial blood pressure

Baseline pre‐intervention values for total cholesterol (TC), triglycerides (TG), high‐density lipoprotein (HDL), very low‐density lipoprotein (VLDL), and low‐density lipoprotein (LDL) were similar between groups (Table 3: *p >* 0.05 for all). There was no effect of beverage condition or time (pre vs. post), nor was there a condition × time interaction for TG, HDL, VLDL, and LDL (Table 3: *p* > 0.05 for all comparisons). A significant condition × time interaction was observed for TC (*p* = 0.03) such that following 2 weeks of consumption of the Goode Health Moroccan Chocolate Blend TC was reduced (Table 3: Pre: 174 ± 26 mg/dL vs. Post: 159 ± 25 mg/dL, *p =* 0.04). Baseline pre‐intervention values for SBP, DBP, and MAP were similar between groups (Table 3: *p* > 0.05 for all comparisons). There was a significant main effect of time for SBP (*p <* 0.001), DBP (*p =* 0.01), and MAP (*p <* 0.01).

## 4 DISCUSSION

This study investigated the impact of consumption of Goode Health Moroccan Chocolate Blend (twice daily for 2 weeks), in a cohort of relatively young and otherwise healthy individuals, on indices associated with cardiovascular health. The primary findings are that endothelial function was improved, and total cholesterol and arterial blood pressure was reduced following the Goode Health consumption. These data provide proof of concept and demonstrate that consumption of Goode Health Moroccan Chocolate blend may serve as an easily administered modifiable lifestyle behavior that can beneficially impact indices associated with improved cardiovascular health.

Reduced endothelial function contributes to elevated risk for and is present in various forms of CVD (Donato et al., 2007; Dow et al., 2017; Gates et al., 2007; Patik et al., 2016; Ramezanzadeh et al., 2023; Rossi et al., 2004; Vanhoutte et al., 2017; Widmer & Lerman, 2014). In the present study, endothelial function, indexed by brachial artery FMD, was elevated following 2 weeks of daily consumption of the Goode Health Moroccan Chocolate Blend in a cohort of relatively young and otherwise healthy individuals. Brachial artery FMD was on average 4.6% at baseline and 5.7% following the beverage consumption phase, which represents an absolute increase of 1.1% and a relative increase of 22% in FMD. Large‐scale studies indicate an approximate 8%–13% reduction in cardiovascular events, in populations with low and high risk, for every 1% increase in absolute brachial artery FMD (Inaba et al., 2010; Matsuzawa et al., 2015; Ras et al., 2013; Thijssen et al., 2019; Xu et al., 2014). Interestingly, this improvement was present despite there being no effect of the Goode Health beverage on baseline absolute brachial artery diameter (Figure 1a) or RH, an index of microvascular function (Figure 1b). While NO bioavailability was not directly assessed, brachial artery FMD is primarily dependent on NO and thus is commonly used to index NO bioavailability (Thijssen et al., 2019); however, the magnitude of NO contribution has been questioned (Wray et al., 2013). Conversely, dilation of the downstream microvasculature occurs through various pathways including those related to prostaglandins, NO, adenosine, various potassium channels, etc. (Rosenberry & Nelson, 2020). Accordingly, our findings suggest that the beneficial impact of the Goode Health beverage may have occurred due to increased NO‐mediated vasodilation of the brachial artery as opposed to an increase in downstream‐mediated microvascular vasodilation. Future studies should confirm this hypothesis using more direct measures of NO bioavailability.

The study did not assess the contribution of each individual ingredient to the measured outcome variables. Many of the ingredients (Table 2) have a beneficial impact on indices associated with cardiovascular health in animal‐based and human studies. Specifically, markers of oxidative stress are reduced following consumption of diets high in or supplementation of soluble fiber (Ghavami, Banpouri, et al., 2023; Ghavami, Ziaei, et al., 2023; Reynolds et al., 2022; Tejani et al., 2023; Theuwissen & Mensink, 2008), natural cocoa (Balzer et al., 2008; Heiss et al., 2005, 2010; Hurr et al., 2015; Jia et al., 2010; Sansone et al., 2015; Schroeter et al., 2006; Sun et al., 2019), green tea (Alexopoulos et al., 2008; Babu et al., 2006; Babu & Liu, 2008), vitamin C (Holowatz & Kenney, 2007; Hurr et al., 2018; Trinity et al., 2016), vitamin D (Harris et al., 2011; Wolf et al., 2020), vitamin E (Nguyen et al., 2021), and folate (Mierzecki et al., 2013; Zamani et al., 2023). Likewise, antioxidative status is elevated and systemic inflammation is reduced following consumption of diets high in or supplementation of these ingredients (Alexopoulos et al., 2008; Babu et al., 2006; Sansone et al., 2015; Schroeter et al., 2006). Mechanistically, these alterations enhance NO bioavailability, which has been linked to improved markers of cardiovascular health including reductions in blood pressure and arterial wall stiffness (Balzer et al., 2008; Heiss et al., 2010; Holowatz & Kenney, 2007; Hurr et al., 2018; Patik, Curtis, et al., 2018; Sansone et al., 2015; Trinity et al., 2016; Zamani et al., 2023). Not surprisingly, these improvements associated with the ingredients contained in the Goode Health Moroccan Chocolate Blend are also linked with improvements in endothelial function when a similar range of these ingredients has been utilized (Balzer et al., 2008; Heiss et al., 2010; Holowatz & Kenney, 2007; Hurr et al., 2018; Patik, Curtis, et al., 2018; Sansone et al., 2015; Trinity et al., 2016; Zamani et al., 2023).

Elevated blood lipids are also a primary culprit in increased risk for and presence of cardiovascular disease (Martin et al., 2024). Indeed, elevated blood lipids contribute to the aforementioned conditions including development and progression of the atherosclerotic process (Martin et al., 2024) and is present in populations with reduced endothelial function (Martin et al., 2024). In the present study, we observed a significant reduction in total cholesterol. As mentioned above, this study was not designed to isolate the contribution of the individual ingredients present in the beverage. However, many of these ingredients are associated with reductions in blood lipids including total cholesterol, low‐density lipoprotein, and triglycerides, as well as elevation in high‐density lipoprotein (Ghavami, Ziaei, et al., 2023; Jia et al., 2010; Mierzecki et al., 2013; Zheng et al., 2011).

Interestingly, the improvements in vascular endothelial function and blood cholesterol were not observed following 2 weeks of consumption of the control beverage. This beverage was comprised of predominately rice protein (65%) and Maltrin M100 maltodextrin (26%) and included trace amounts of flavoring from monk fruit, stevia, vanilla, and caramel flavors. Importantly, the control beverage did not contain all of the ingredients that are present in the Goode Health Moroccan Chocolate Blend beverage, such as bilberry, isoquercetin, turmeric, bergamot, berberine, ginger, black garlic, natural cocoa, green tea, and more, which have high polyphenol, antioxidant, and anti‐inflammatory properties. Previous studies have reported that products with reduced polyphenol and anti‐inflammatory compounds result in reduced antioxidant and anti‐inflammatory properties (Kang et al., 2010; Sawicki et al., 2024), reduced protection against biomarkers associated with elevated CVD risk (Drewnowski & Gomez‐Carneros, 2000; Kang et al., 2010), and elevated risk for other complications including elevated cancer risk, metabolic complications, and microbiome/gut issues (Drewnowski & Gomez‐Carneros, 2000; Prescott et al., 2023; Sanchez‐Terron et al., 2024). Accordingly, it is likely that the divergent responses between the two beverage conditions are related to the control beverage, which was a vegetable protein blend that did not contain many of these ingredients, particularly those with polyphenol and anti‐inflammatory properties.

### 4.1 Methodological considerations

As previously mentioned, the goal was to evaluate the impact of a commercially available beverage, designed as an easily administered lifestyle intervention. As a result, we are not able to determine the impact of the specific ingredients contained within the beverage. While there was a range of age, BMI etc. among the participants (Table 1) they were all otherwise healthy and free of known cardiovascular, metabolic, or neurocognitive disease. We believe this strengthens the findings because it suggests that the beneficial impact can be observed in a middle‐aged population (average: 33 ± 12 years) prior to the onset of overt disease. Future studies should investigate the beverage's benefit on elderly individuals and populations with elevated risk for, or the presence of, overt diseases such hypertension, type II diabetes, hypercholestermia etc. In addition, while all participants self‐reported being recreationally active we did not directly assess aerobic fitness. Likewise, we did not assess dietary behaviors of the individuals. Given that physical fitness and dietary habits, particular in food items that are well known to increase NO production (i.e., leafy greens, beats, garlic, unprocessed dark chocolate etc.) can influence baseline vascular function as well as influence responsiveness to various dietary interventions, it is important to consider these variables in future studies. That being said, all individuals were asked to maintain their normal daily physical activity and dietary behaviors during the intervention periods, and thus it is unlikely that the observed findings are related to alterations in lifestyle other than the consumption of the study beverages. The study sample includes both male and female participants in effort to broaden the “real‐life” applicability. As a result, we are not sufficiently powered to determine if there are differing responses in males versus females or if there is an influence of contraception use/type. Future studies should consider these variables as well. Lastly, while the observed beneficial impacts after 2 weeks are noteworthy, future studies should explore whether continued or augmented benefits occur with longer intervention periods. While 83% compliance is good, this approach would also allow for assessment of the sustained compliance and feasibility and acceptability of this intervention during a longer intervention period.

## 5 CONCLUSION AND PRACTICAL SIGNIFICANCE

These findings indicate a beneficial impact of 2 weeks of consumption of the Goode Health Moroccan Chocolate Blend on well‐validated indices associated with cardiovascular health: blood lipid profile, arterial blood pressure, and vascular endothelial function. This is particularly impressive given that these improvements were observed following a relatively short‐duration of 2 weeks. Importantly, improvements in blood pressure, particularly systolic blood pressure, as well as blood lipids significantly reduces risk for the development of coronary heart disease or CVD over a 10‐year period (D'Agostino Sr. et al., 2008). Ultimately, these data suggest that Goode Health Moroccan Chocolate Blend consumption improves indices of physiological health and may be a useful and easily administered lifestyle modification intervention to improve critical indices of physiological health.

## AUTHOR CONTRIBUTIONS

J.O.K. contributed to the study design, data analysis, data interpretation, and drafting of this manuscript. R.M.B. contributed to the study design, data collection, data analysis, data interpretation, and drafting/editorial process of the manuscript. All authors approved the final version of this manuscript.

## FUNDING INFORMATION

This study was supported by Goode Health LLC (RMB). The company was not involved in experimental design, analysis and interpretation of results, or preparation of the manuscript.

## CONFLICT OF INTEREST STATEMENT

The authors have no conflict(s)‐of interest or disclosures to report.

## ETHICAL STATEMENT

Ethical approval was granted by the Institutional Review Board at the University of Texas at Artlington.

## Data Availability

All raw data were collected and generated at the University of Texas at Arlington. Data supporting the findings and conclusions of this study are available from the corresponding author upon reasonable request.
